# Genome-Wide Polymorphism and Comparative Analyses in the White-Tailed Deer (*Odocoileus virginianus*): A Model for Conservation Genomics

**DOI:** 10.1371/journal.pone.0015811

**Published:** 2011-01-19

**Authors:** Christopher M. Seabury, Eric K. Bhattarai, Jeremy F. Taylor, Ganesh G. Viswanathan, Susan M. Cooper, Donald S. Davis, Scot E. Dowd, Mitch L. Lockwood, Paul M. Seabury

**Affiliations:** 1 Department of Veterinary Pathobiology, College of Veterinary Medicine, Texas A&M University, College Station, Texas, United States of America; 2 Division of Animal Sciences, University of Missouri, Columbia, Missouri, United States of America; 3 Texas AgriLife Research, Texas A&M University System, Uvalde, Texas, United States of America; 4 Research and Testing Laboratories, SpiroStat Technologies, Medical Biofilm Research Institute, Lubbock, Texas, United States of America; 5 Texas Parks and Wildlife Department, Kerrville, Texas, United States of America; 6 ElanTech, Inc., Greenbelt, Maryland, United States of America; Royal Tropical Institute, The Netherlands

## Abstract

The white-tailed deer (*Odocoileus virginianus*) represents one of the most successful and widely distributed large mammal species within North America, yet very little nucleotide sequence information is available. We utilized massively parallel pyrosequencing of a reduced representation library (RRL) and a random shotgun library (RSL) to generate a complete mitochondrial genome sequence and identify a large number of putative single nucleotide polymorphisms (SNPs) distributed throughout the white-tailed deer nuclear and mitochondrial genomes. A SNP validation study designed to test specific classes of putative SNPs provides evidence for as many as 10,476 genome-wide SNPs in the current dataset. Based on cytogenetic evidence for homology between cow (*Bos taurus*) and white-tailed deer chromosomes, we demonstrate that a divergent genome may be used for estimating the relative distribution and density of *de novo* sequence contigs as well as putative SNPs for species without draft genome assemblies. Our approach demonstrates that bioinformatic tools developed for model or agriculturally important species may be leveraged to support next-generation research programs for species of biological, ecological and evolutionary importance. We also provide a functional annotation analysis for the *de novo* sequence contigs assembled from white-tailed deer pyrosequencing reads, a mitochondrial phylogeny involving 13,722 nucleotide positions for 10 unique species of Cervidae, and a median joining haplotype network as a putative representation of mitochondrial evolution in *O. virginianus*. The results of this study are expected to provide a detailed template enabling genome-wide sequence-based studies of threatened, endangered or conservationally important non-model organisms.

## Introduction

Organisms traditionally considered of “minor” importance by national and international funding agencies generally suffer from a paucity of genome-wide sequence and polymorphism data which severely limits the implementation of genomic approaches for addressing biological questions in these species. One such underserved species is the white-tailed deer (*Odocoileus virginianus*), a highly successful and widely distributed ruminant mammal species of the order Artiodactyla and family Cervidae [1]–[3]. Within the genus *Odocoileus*, the primary species are the white-tailed deer (*O. virginianus*) and mule deer (*O. hemionus*; for review see [2], [3]), with these species possessing equivalent karyotypes (2n = 70) [4].

Based primarily on geographic variation in body size, differences in antler growth, and other relatively minor morphological characteristics, as many as 38 subspecies of *O. virginianus* have been suggested, including the endangered Florida key deer (*O. virginianus clavium*), and Columbian white-tailed deer (*O. virginianus leucurus*) [1], [3]. Currently, free ranging white-tailed deer are ubiquitously distributed throughout most of the United States, with representative populations extending from Canada to Mexico, Central America, and South America [2], [3], [5]. Moreover, white-tailed deer have adapted to a variety of landscapes and environmental conditions while also exhibiting exceptional potential for recruitment [6]–[8], and despite historic overexploitation, appear to possess relatively high levels of genetic diversity [7], [9], [10].

Unlike most traditional model organisms, and many free ranging wildlife species, white-tailed deer are distributed across a large range that includes both free ranging and captive populations, with a recent trend in North America toward establishing captively-propagated livestock lines from founders originating from a variety of wild populations. The farming of deer, and particularly white-tailed deer, has become a significant alternative livestock industry within the U.S. and Canada [11], [12]. Consequently, U.S. captive and free-ranging populations of white-tailed deer have been subjected to intense surveillance for a variety of infectious diseases, with considerable efforts expended towards disease prevention and control [11]–[18]. Despite the growing numbers of captive and free ranging white-tailed deer under active management or surveillance in the U.S., little information exists concerning genomic diversity and divergence among populations. Previous genetic studies have primarily focused on genetic diversity, parentage, or the evaluation of population structure using relatively few microsatellite loci, with many of these markers derived from the domestic cow or sheep [7], [9], [10], [19], [20]. Recent research has targeted associations between naturally occurring polymorphisms within candidate genes and susceptibility to chronic wasting disease, a fatal transmissible spongiform encephalopathy (TSE) known to affect several species of Cervidae [21]–[24]. However, the inability to apply genome-wide approaches to address questions related to deer biology and evolution has severely limited progress. Thus, a substantial need exists to develop cost-effective *de novo* approaches which will rapidly enable sophisticated research programs for biologically important species for which research funds are limited.

We generated a reduced representation library (RRL) [25] to reduce the complexity of the white-tailed deer genome and a random shotgun library (RSL) to enable massively parallel pyrosequencing via the Roche 454 platform. The resulting sequences were assembled using a *de novo* approach, and contig alignments were used to identify a large number of putative single nucleotide polymorphisms (SNPs) distributed throughout the nuclear and mitochondrial genomes. Herein we also produced a complete mitochondrial genome sequence assembly for the white-tailed deer, with annotations supported by comparative sequence analysis, and a Bayesian mitochondrial phylogeny involving 10 unique species of Cervidae. Validated mitochondrial SNP variation and a median joining haplotype network analysis were utilized to investigate mitochondrial evolution in *O. virginianus*. Based upon established homology between domestic cow (*Bos taurus*) and white-tailed deer chromosomes [4], we utilized a method of comparative contig overlay with the *B. taurus* genome assembly to estimate the genomic distribution and relative density of white-tailed deer contigs and putative SNPs. Finally, we conducted a functional annotation analysis to characterize and classify the genomic information content of contigs produced from the *de novo* assembly of the pyrosequencing data. Our results clearly demonstrate that species-specific *de novo* assemblies in conjunction with comparative contig overlay can be used to enable whole-genome analyses for species with little or no genome sequence data. Moreover, we also utilize novel genome-wide sequence data and reagents to produce the first large-scale genome-wide polymorphism and comparative analyses for *O. virginianus*. The results of this study will enable genomics research for all species of *Odocoileus*, including the endangered Florida key deer, and will facilitate efforts to identify genetic variation associated with health-related trait information, genome-wide signatures of selection, and genomic variation underlying mechanisms of adaptation.

## Results and Discussion

### White-tailed Deer RRL Analysis

A white-tailed deer RRL was constructed using 16 unrelated individuals representing northern and southern U.S. nuclear germplasm via *Alu*I restriction enzyme digestion of a pooled DNA sample followed by a manual reduction in genome complexity via gel excision and purification of size-selected (≈350–400 bp) DNA fragments [26]. Collectively, 1,206,716 high quality reads comprising 285,269,784 bp of sequence were produced from this RRL on a Roche GS FLX instrument. A three stage assembly procedure employing strict requirements for read matching (sequence read length fraction = 0.90; similarity = 0.90) was used to create a reference assembly while also discouraging the creation of false contigs. The analytical workflow included an initial *de novo* assembly of RRL sequence reads, repeat masking of the resulting contigs, and utilization of the masked contig sequences to perform a reference assembly using the RRL sequencing reads (CLC Genomics Workbench 3.7.1). The resulting assembly contained 55,526 contigs comprising 19,207,189 bp of nucleotide sequence, with an average contig length of 346 bp. The minimum estimated repetitive DNA content for the 55,526 contigs was approximately 17%, as predicted by RepeatMasker (Human and/or Bovine Repeat Libraries). This relatively low estimate reflects our inability to mask all white-tailed deer repeats given the absence of a complete species-specific repeat library. Utilization of the masked contigs to perform a RRL reference assembly produced 44,385 final contigs averaging 338 bp, with approximately 6.2 sequence reads/contig, and a mean depth of 4.2X (Table S1). However, more than 95% of all contigs possessed <4X coverage (see Table S1 for coverage distribution), and when contigs possessing ≥20X coverage were excluded, the mean depth was approximately 2.1X (SD = 1.31). Unmasked repeats and/or potential copy number variants were apparent based on the observed depth of coverage achieved for the final contigs (see Table S1), with 392 contigs that possessed ≥20X coverage. However it is also likely that some repeats and/or copy number variants are present in contigs possessing lower coverage. Therefore, genomic sequence information derived from our RRL contigs will contribute to establishing an annotated white-tailed deer repeat library, and may also help elucidate potential copy number variants.

Alignment of the final white-tailed deer RRL contigs to the bovine genome sequence assembly (Btau4.0) via blastn resulted in 18,301 contigs producing 19,667 E-value informative hits (E-value≤1e-50) to either a single chromosome (BTA1-BTAX; MT; discrete unknown, chrUN; (≤3 chromosomal positions) or a single chromosome plus one unknown chromosome (≤3 chromosomal positions). These alignment criteria were chosen to maximize the likelihood of achieving unambiguous alignments while also allowing for potential gene family members, duplications, and limitations of the bovine genome assembly (i.e., assembly errors, chrUn unassigned sequence contigs). Overall, the average percent identity was approximately 92%, with an average alignment length of 306 bp, and 17,084 contigs (93%) produced one unique alignment to a bovine chromosome (Table S2).

Collectively, 6,877 putative SNPs (6,724 diallelic; 153 with >2 alleles) were detected within 18,301 blastn-aligned contigs using a 3X minimum depth of coverage for all potential variable sites (Table S3), with 5,710 (83%) putative SNPs derived from 17,084 contigs that produced one unique blastn hit. The average estimated minor allele frequency (MAF) for the 6,724 diallelic SNPs was 0.282. The distribution of blastn hits (n = 19,667) for all aligned contigs (n = 18,301) and putative SNPs (n = 6,877) against the bovine genome is shown in Figure S1, with similar results for the 17,084 uniquely aligned contigs and 5,710 putative SNPs depicted in Figure S2. The average deer-to-bovine hit density was one deer contig every 142.7±27.7 kb. Absence of BTAY annotation precluded Y-specific comparative contig overlays between *B. taurus* and white-tailed deer.

Interestingly, we observed a disproportionately large number of SNPs for deer contigs that aligned with BTA28. Further investigation revealed two clusters comprising 14 total contigs that aligned to BTA28 as follows: 1) Between *CHRM3* and *ZNF33B* (11.35–11.39 Mb; n = 12 contigs); and 2) Within a putative intronic region of *LOC532077* (11.620918–11.620982 Mb; n = 2 contigs). Both bovine regions are near a small break in the cattle-human comparative map [27] that is also proximal to the HSA10 centromere. Furthermore, this region of BTA28 is near the break between previously described HSA1 and HSA10 homologous synteny blocks [27]. Collectively, these 14 deer contigs contained 424 putative SNPs (Table S2), and may represent a duplicated and/or expanded region of the white-tailed deer genome that also serves as a break point between cattle and white-tailed deer chromosomes. Interestingly, the homologue of BTA28 has not yet been identified in *Odocoileus* species, with both white-tailed and mule deer possessing five more autosomes than cattle [4].

To functionally characterize the sequence content of 18,301 blastn-aligned contigs, we performed functional annotation, pathway mapping, and putative ortholog matching by mapping the assembled sequences onto relevant classification schemes such as Gene Ontology (GO) terms [28], KEGG pathways [29], and Swiss Prot Protein keywords [30] using both Krakenblast [31] and the Database for Annotation, Visualization, and Integrated Discovery (DAVID) [32]. Despite the relatively low genomic sequence coverage, 1,801 contigs (9.8%) produced functional hits and detailed annotation data, with 322 functional ontology terms, categories, and keywords cumulatively identified (Table S4).

### Pooled White-tailed Deer RRL and RSL Analysis

In addition to the RRL, we constructed a random shotgun library (RSL) from a single male deer included in the RRL and produced 778,792 sequence reads comprising 286,843,168 bp on a Roche GS FLX instrument. The pooled RRL and RSL reads (n = 1,985,508) were assembled via CLC Genomics Workbench 3.7.1 using the previously described three stage *de novo* assembly process, which resulted in 126,980 contigs (average contig length of 433 bp) representing 55,020,760 bp of sequence. The minimum estimated repetitive content predicted by RepeatMasker was approximately 21% (Human and/or Bovine Repeat Libraries). After masking the *de novo* contigs and performing a reference assembly, 94,070 final contigs averaging 400 bp, with approximately 5.0 reads/contig, and a mean depth of 3.4X remained (Table S5). Nevertheless, more than 95% of all contigs possessed <5X coverage (See Table S5 for coverage distribution), and when contigs possessing ≥20X coverage were excluded, the mean depth was approximately 2.1X (SD = 1.45). Examination of the distribution of coverage across all final contigs provided evidence for unmasked repeats as well as potential copy number variants, with 536 contigs that possessed ≥20X coverage. However, some unmasked repeats and/or copy number variants are also likely to be present at lower depths of coverage, with outliers visualized by plotting coverage across all contigs (plot not shown; see Table S5). Final contig alignment to the bovine genome (Btau4.0) via blastn resulted in 56,084 contigs producing 61,553 hits (E≤1e-50) to either a single bovine chromosome (≤3 chromosomal positions), or a single chromosome plus one chrUN (≤3 chromosomal positions). Average percent identity was approximately 92%, with an average alignment length of 384 bp, and 51,087 contigs (91%) produced a unique alignment to a single chromosome (Table S6).

**Figure 1:**
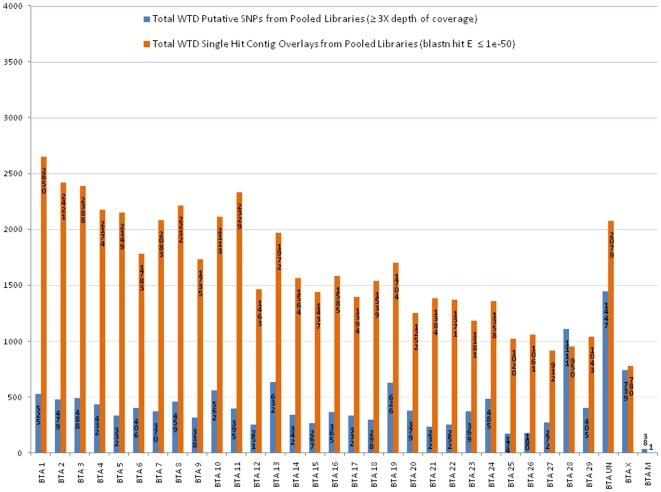
Comparative Contig Overlay with Putative SNPs. Histogram displaying the bovine chromosome locations (Btau4.0) of 51,087 uniquely aligned (E≤1e-50) white-tailed deer (WTD) sequence contigs and 13,962 putative SNPs (≥3X coverage) derived from sequencing a WTD reduced representation library (RRL) and random shotgun library (RSL; Also See Table S6, S7, and S8). Note, one WTD contig that aligned to BTA13 (Consensusfrom53142contig) was removed by NCBI filtering.

From the pooled analysis we detected 17,813 putative SNPs (17,266 diallelic; 547 with >2 alleles) within 56,084 blastn-aligned contigs using a 3X minimum depth of coverage requirement for potential variable sites (Tables S7, S8). Notably, 13,962 (78%) putative SNPs were located within contigs which aligned uniquely to the bovine genome. Nevertheless, some caution is necessary when interpreting this result given the possibility for at least some closely linked multicopy loci for which only a single representative was incorporated into a relevant bovine chromosomal assembly. Average estimated MAF for the 17,266 diallelic SNPs predicted was 0.274. The distribution of blastn hits (n = 61,553) for all aligned contigs (n = 56,084) and putative SNPs (n = 17,813) with respect to the bovine genome are shown in Figure S3. A plot for the 51,087 uniquely aligned contigs and corresponding 13,962 putative SNPs is displayed in Figure 1. For the pooled analysis (RRL+RSL), the average deer-to-bovine hit density was one deer contig every 45.9±9.9 kb. Similar to the RRL only analysis, we again observed a disproportionately large number of putative SNPs predicted for 27 white-tailed deer contigs that aligned to the same two regions of BTA28. In total, those 27 contigs contained 900 putative SNPs (Table S7).

In the RRL analysis, only six contigs provided evidence for a putative mitochondrial origin (Figure S1). However, our pooled RRL and RSL analysis effectively captured the complete white-tailed deer mitochondrial genome (GenBank Accession HQ332445) at an average coverage of 70.4X, with 2,965 reads included in the final assembly. Using the *B. taurus* and caribou (*Rangifer tarandus*) mitochondrial genome refseqs (GenBank accessions NC_006853.1; NC_007703) in conjunction with BLAST (blastn, bl2seq, blastp; http://blast.ncbi.nlm.nih.gov/), we successfully annotated 13 white-tailed deer mitochondrial protein coding genes (*ND1*, *ND2*, *COX1*, *COX2*, *ATP8*, *ATP6*, *COX3*, *ND3*, *ND4L*, *ND4*, *ND5*, *ND6*, *CYTB*) and two ribosomal RNA genes (*12S*, *16S*; GenBank Accession HQ332445). Using tRNAscan-SE (http://lowelab.ucsc.edu/tRNAscan-SE/) [33], we also predicted 21 tRNA genes (GenBank Accession HQ332445). The consensus mitochondrial genome spanned 16,477 contiguous bp and possessed an average GC content of 36.9%. In total, 38 putative diallelic SNPs were detected with ≥3X coverage (average coverage 78X) and an overall average estimated MAF of 0.07 (Table S8). A blastn query of the white-tailed deer mitochondrial genome sequence against the nucleotide collection (nr/nt) using BLAST (http://blast.ncbi.nlm.nih.gov/Blast.cgi; blastn) produced a top hit (based on 100% coverage; 93% Max Identity) to the complete mitochondrial genome of *R. tarandus* (GenBank Accession NC_007703).

To functionally characterize the sequence content of the 56,084 blastn-aligned contigs, the assembled sequences were again mapped onto the relevant classification schemes, with 5,057 contigs (9%) producing putative functional hits and detailed annotation data, including 606 identified functional ontology terms, categories, and keywords (Table S9). Pooling sequences obtained from the RRL and RSL resulted in a >2.8 fold increase in the total number of contigs predicted to possess at least partial gene sequences, thereby elucidating nucleotide sequence data for as many as 3,097 putative genes (see unique hits Table S9).

### SNP Validation and Mitochondrial Evolution

**Figure 2:**
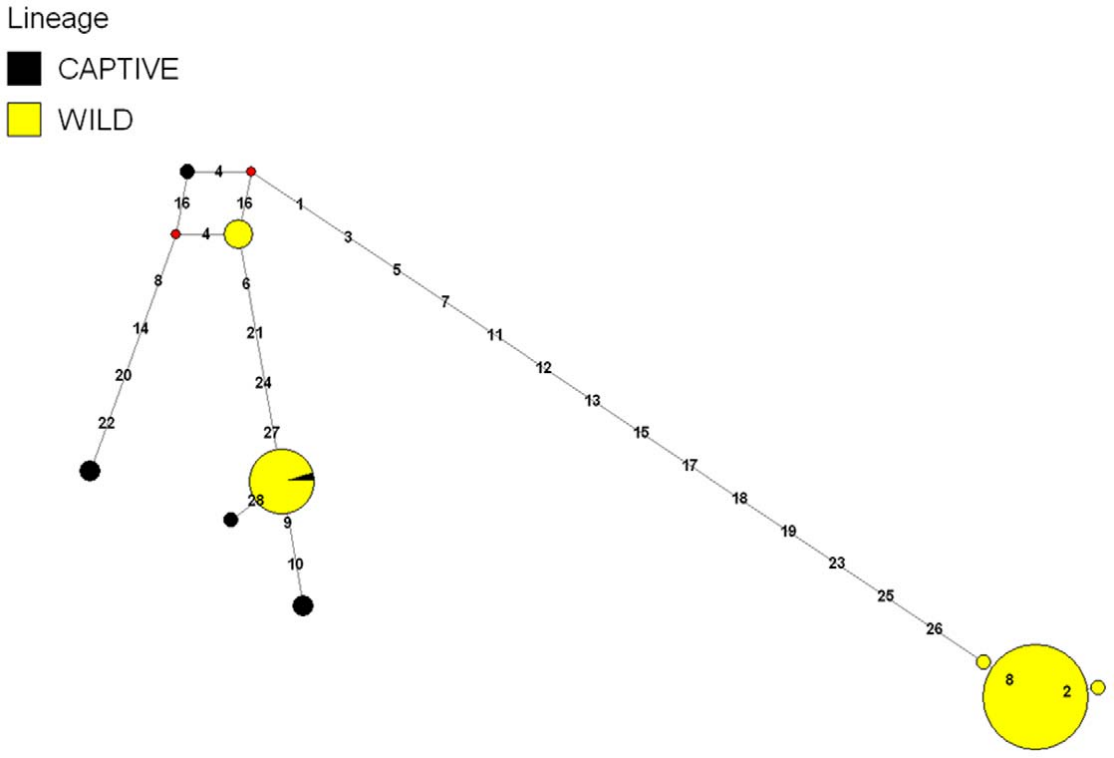
Mitochondrial Haplotype Network. Median joining (MJ) haplotype network for white-tailed deer mitochondrial SNP variation (n = 28 validated SNPs) discovered by sequencing reduced representation and random shotgun libraries. Numbers indicate SNP positions in numerical order (Table S8). Node sizes are proportional to haplotype frequency, and all branch lengths are drawn to scale. Median vectors are indicated by small red circles. Nine discrete haplotypes were detected among samples from one free-ranging population (yellow) and representatives from three captive populations (black).

**Figure 3:**
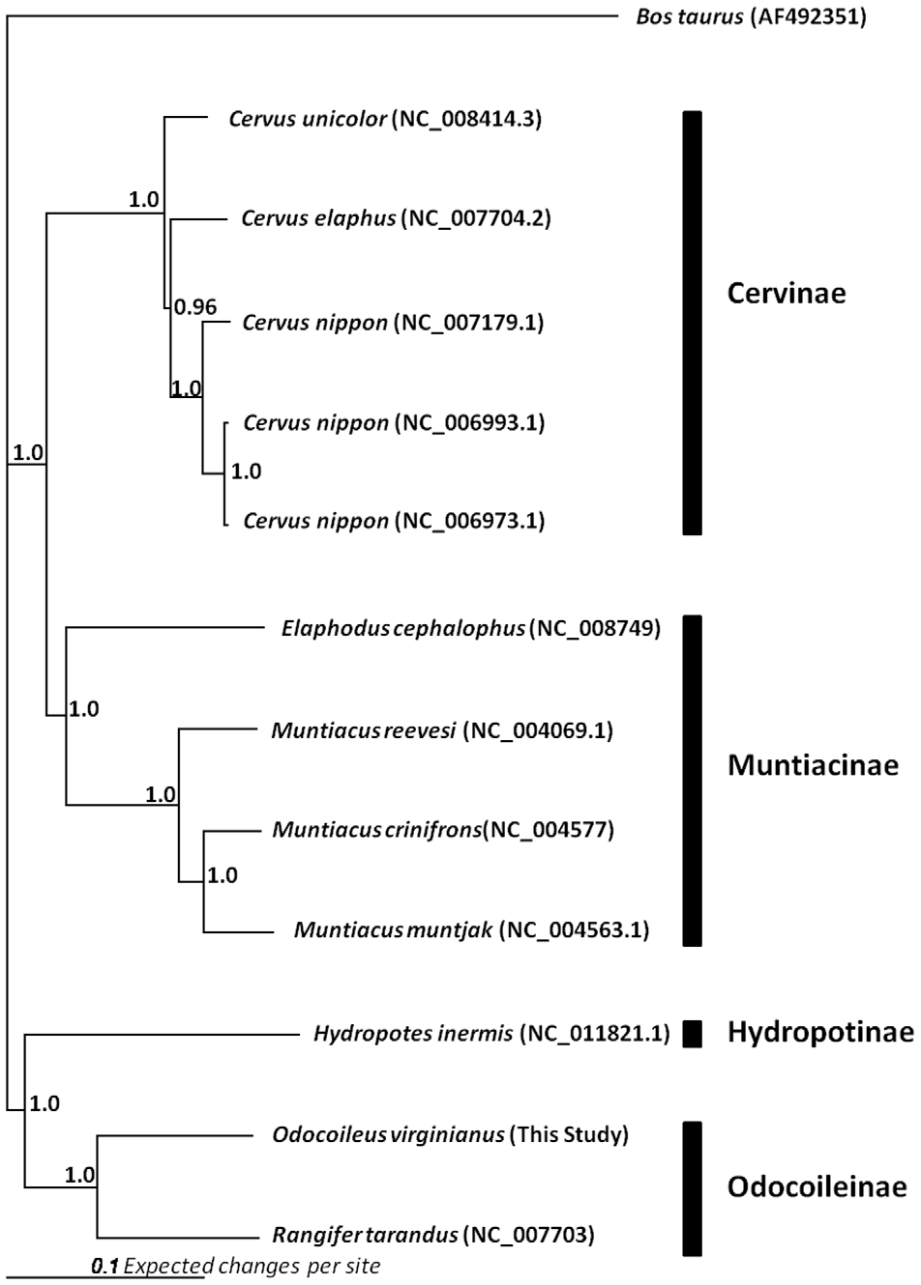
Mitochondrial Phylogeny. Majority rule consensus phylogenetic tree for 10 species of Cervidae inferred from concatenated *12S rRNA*, *16S rRNA*, *ND1*, *ND2*, *COX1*, *COX2*, *ATP8*, *ATP6*, *COX3*, *ND3*, *ND4L*, *ND4*, *ND5*, *ND6*, and *CYTB* gene sequences. Entries in parenthesis represent Genbank Accession Numbers. The *Odocoileus virginianus* consensus sequence was derived from this study. Start codons, stop codons, and overlapping regions of genes were excluded. Numbers beside branches indicate posterior probabilities estimated from a Bayesian analysis employing 2×10^6^ generations [35].

Using a DNA panel of 96 white-tailed deer that included the 16 deer used to create the RRL and RSL, we estimated the proportion of total putative SNPs that were likely to be valid by fluorescent allele-specific PCR [34]. Putative nuclear SNPs were sampled from a range of discovery classes defined by differences in depth of coverage and observed minor allele count (Table 1), with efforts to avoid clustered SNPs and those predicted within or proximal to homopolymers. Every putative SNP detected within the white-tailed deer mitochondrial genome was also tested (n = 38). SNP validation provided evidence for as many as 10,448 nuclear SNPs (Table 1) and 28 mitochondrial SNPs (Table S7, S8). Lower validation rates for mitochondrial SNPs are likely a reflection of two primary issues: 1) Not all sequence compositions, including but not limited to tightly clustered SNPs, are amenable to genotyping by allele specific KASPar assays [34]; and 2) Some of the putative mitochondrial SNPs queried were predicted in regions that were rich in one or two specific nucleotides (including homopolymers), which likely increased local read error. Median joining haplotype networks constructed for both captive and free-ranging white-tailed deer revealed extensive mitochondrial haplotype variation and divergence (Figure 2). Using the white-tailed deer mitochondrial genome consensus sequence, we implemented a Bayesian approach [35] to estimate the evolutionary history for 10 species of Cervidae (Figure 3). Collectively, 13,722 nucleotide positions spanning the *12S rRNA*, *16S rRNA*, *ND1*, *ND2*, *COX1*, *COX2*, *ATP8*, *ATP6*, *COX3*, *ND3*, *ND4L*, *ND4*, *ND5*, *ND6*, and *CYTB* genes were used to produce the phylogeny. Distance, maximum composite likelihood, and maximum parsimony approaches (transitions+transversions) employing bootstrap resampling produced similar overall tree topologies, with the exception that *Elaphodus cephalophus* and *Hydropotes inermis* could not be unambiguously placed within the tree due to inadequate bootstrap support (bootstrap≤70; trees not shown). Our Bayesian mitochondrial phylogeny (Figure 3) provides strong support for monophyly in Cervinae, Muntiacinae, and Odocoileinae for the surveyed taxa while also providing evidence that the single species representing the subfamily Hydropotinae (*H. inermis*) is more closely related to Odocoileinae (new world deer) than any other subfamily represented in our analysis. These findings are generally consistent with recent phylogenies produced from either partial mitochondrial genome sequences [36]–[38] and/or the hybrid combination of nuclear and mitochondrial sequence information [39]. Additionally, the position of *O. virginianus* within our tree is consistent with a nuclear phylogenomics study involving 39,695 parsimony informative characters derived from 40,843 nuclear SNPs [40]. Our ability to produce a robust mitochondrial phylogeny indicates that similar sequencing approaches in other non-model organisms are also likely to produce massive amounts of phylogenetically informative data.

**Table 1:** Summary data for a white-tailed deer RRL plus RSL SNP validation study.

SNP Coverage Class	Minor Allele Count	Total Putative SNPs	% SNPs^1^	Total SNPs Tested	Assays Passing Q.C.^2^	Total SNPs Validated (%)	Total SNPs Predicted^3^
3X	1	6791	51.9%	15	14	14 (100)	6791
4X	1	1951	14.9%	15	13	13 (100)	1951
4X	2	497	3.8%	12	12	12 (100)	497
5X	1	778	5.9%	12	12	10 (83.3)	648
5X	2	255	1.9%	12	9	7 (77.8)	198
6X	1	467	3.6%	12	9	7 (77.8)	363
Totals/Avg		10,739	82.0%	78	69	63 (89.8)	10,448^4^

1 Percent of all nuclear SNPs based on total putative SNPs with 3X–150X coverage predicted within 51,087 single-hit contigs (E≤1e-50 to a unique bovine genomic position).

2 Allele-specific SNP assays exhibiting acceptable and repeatable genotype clustering.

3 Prediction based on the proportion of validated nuclear SNPs sampled from each class.

4 Mitochondrial validation assays also confirmed 28 additional SNPs (28/38; 74%).

In addition to our phylogenetic analyses, we also aligned the annotated white-tailed deer mitochondrial sequence with known bovine mitochondrial polymorphism data using blastn. The results of our alignments revealed one validated SNP that was conserved among highly divergent taxa. Specifically, nucleotide 633 of the predicted *CYTB* gene for the white-tailed deer (10481Y; (Codon 211; Ile→Ile); Table S8) was also variable (C/T) in at least four lineages of *Bos*, including *B. taurus*, *B. indicus*, *B. frontalis*, and *B. javanicus* (Genbank Accessions HM045018.1, EU177870.1, EF685907.1, AY079130.2, AB542189.1, FJ556556, AY689188.1, FJ997262.1). Importantly, amino acid 211 of the *CYTB* gene falls within a domain of unknown function according to the Simple Modular Architecture Research Tool online (http://smart.embl-heidelberg.de/). The unusual nature of this shared synonymous polymorphism provides some potential evidence for either selective and/or functional constraint at amino acid 211 across several highly divergent taxa and should be further investigated.

### Prospects for a complete white-tailed deer genome sequence

Generation of a white-tailed deer genome sequence assembly would provide a valuable resource for population geneticists and physiologists studying the basis of adaptation and fecundity [5]–[8]. In addition to interesting biological characteristics, burgeoning U.S. white-tailed deer populations may potentially become reservoirs for infectious disease, and also increase the likelihood for deer-human conflict [8], [41]–[44]. Importantly, the majority of emerging infectious diseases in humans are zoonotic, with most originating from wildlife populations which also serve as vectors for nonzoonotic diseases affecting livestock (for review see [44]–[47]). The availability of a genome sequence would directly enable population-based studies of host-disease relationships while also providing a genomic basis for modern management strategies.

At a cost of $10,000 per GS FLX instrument run, with each fully optimized run producing approximately 400 Mb of sequence data, a theoretical 1X coverage of the white-tailed deer genome would cost approximately $70,000, assuming a genome size similar to that of the domestic cow (*B. taurus*, 2.87 Gb genome; [48]). Moreover, a hybrid combination of currently available long (Roche GS FLX) and short read sequencing technologies (Illumina or Applied Biosystems) would generate sufficient sequence data from mate-pair and paired-end libraries to allow a *de novo* white-tailed deer assembly at a modest cost, with single molecule sequencing [49] soon to further reduce the cost of *de novo* genome assemblies. However, every genome sequence needs a good map [50], and the SNPs identified in this study will enable linkage and/or linkage disequilibrium approaches to producing a robust physical and/or genetic map capable of guiding the white-tailed deer sequence assembly.

### Conclusions and Future Studies

This study demonstrates that small, highly targeted investments in sequencing for “minor” species can rapidly enable genomic and phylogenomic research programs via adaptation of bioinformatic procedures developed for human or well-funded food animal species [25], [26], [51], [52]. We have generated a substantial number of high quality white-tailed deer nuclear sequence contigs which were unambiguously aligned to the bovine genome assembly despite 32.5 million years of species divergence, as predicted from a mitochondrial *CYTB* and *16S* rRNA analysis [53]. Our relatively small, targeted sequencing initiative lead to the generation of a complete white-tailed deer mitochondrial genome sequence and phylogeny while also providing a new resource for population-based studies and genetic discrimination analyses for *Odocoileus* species. The mitochondrial genome sequence, nuclear genome sequence contigs, putative and validated SNPs, and functional annotation will directly facilitate conservation, population, management, and genetic epidemiology research in *O. virgianus* and *O. hemionus* while also enabling conservation genomics in the endangered Florida Key deer.

## Materials and Methods

### Construction and Sequencing of the RRL and RSL

In the absence of a genome sequence against which to perform an *in silico* whole genome digestion [51], a molecular prediction based upon a domestic animal species was employed. Using knowledge from the construction of a pig (*Sus scrofa*) RRL [26], two restriction enzymes (*Alu*I; *Hae*III; New England Biolabs) which recognize four base sequences (AG|CT; GG|CC) and produce blunt-ended fragments were tested via overnight digestion of 2.8 µg of white-tailed deer DNA (*O. virginianus*; 37°C; 8 U enzyme/µg DNA). *Alu*I was selected for RRL preparation based on modest repetitive content observed within size-selected swine digestion fragments [26], its apparent ability to fully digest white-tailed deer DNA, presence of the restriction site in deer mitochondrial sequences (Genbank accessions U12869.1; M35874.1), and the paucity and distribution of visually apparent bands following digestion (Figure S4).

Equal amounts of DNA were pooled from 16 (n = 7 females; n = 9 males) unrelated white-tailed deer representing a sampling of northern and southern U.S. nuclear germplasm derived from both captive and free ranging populations. Ethical clearance is not applicable to samples obtained from lawfully harvested white-tailed deer. A total of 14 µg of DNA was digested overnight (37°C; 8 U enzyme/µg DNA) with *Alu*I, as suggested by the manufacturer (New England Biolabs). The resulting fragments were separated via 2.5% ULTRA-SIEVE high resolution agarose (IBI Scientific) gel electrophoresis according to instructions for enhanced separation (IBI Scientific), with ethidium bromide staining for visualization. Three regions of the gel were excised for purification: A) Fragments ≈350–400 bp, with efforts to avoid a faint band at ≈340 bp; B) Fragments ≈220–280 bp; and C) Fragments ≈160–180 bp. Restriction fragments were purified using the Qiaquick Gel Extraction Kit (Qiagen), with an extended elution incubation period (10–12 min) and reduced elution volume (20 µl). Following gel purification, the smaller fragments were archived and the largest fragment population (≈350–400 bp) was utilized for pyrosequencing (Figure S4). A sequencing library was constructed via random ligation of sequencing adaptors provided with the GS FLX titanium library kit (Roche Applied Science). All library preparation, emulsion PCR, quantitation, and sequencing steps followed manufacturer protocols (Roche Applied Science).

To construct a white-tailed deer RSL, one male white-tailed deer (*O. virginianus*) utilized in the preparation of the RRL was selected and a GS FLX Titanium Rapid Library was prepared via nebulization, fragment end repair, random ligation of sequencing adaptors, fragment size selection, and quantitation as directed by the manufacturer (Roche Applied Science). All subsequent procedures also followed manufacturer protocols (Roche Applied Science).

### Sequence Assembly, SNP Detection, BLAST annotation, and tRNA Prediction

Two assemblies, each with SNP detection and BLAST annotation analyses were performed using identical workflows. For the first assembly, only sequence reads generated from the RRL were utilized (RRL only), whereas the second assembly included sequence reads from both the RRL and the RSL (Pooled RRL+RSL). In both cases, a three step assembly procedure was utilized in conjunction with CLC Genomics Workbench 3.7.1 (CLC Bio) and RepeatMaster (http://www.repeatmasker.org/). Unmasked sequence reads were imported into CLC Genomics Workbench 3.7.1 and a strict *de novo* assembly was performed with user defined parameters: add conflict annotations = yes, conflict resolution = vote (A,T,C,G), create report = yes, create sequence list = yes, non-specific matches = ignore, minimum contig length = 200 bp, mismatch cost = 2, insertion cost = 3, deletion cost = 3, minimum read length fraction = 0.90, minimum fraction of identity (similarity) = 0.90. Contigs produced were processed with RepeatMasker (http://www.repeatmasker.org/; RepBase15.0.2), and the masked contigs became the reference sequences used for SNP discovery; an approach similar to that used for the rainbow trout [52]. Masked contig reference sequences were exported for subsequent filtering by local blastn searches against Btau4.0 (E≤1e-50) and scenario specific (RRL only; RRL+RSL) SNP detection analyses. Custom scripts were engineered to parse contig assignments to bovine chromosomes as follows: E-value informative hit to BTA1-BTAX; MT; discrete chrUN; (≤3 chromosomal positions); to a single chromosome plus one discrete chrUN (≤3 chromosomal positions), and to a unique chromosomal location. The distribution and density of all contig alignments were examined relative to their predicted locations within the bovine genome (Btau4.0).

SNP detection analyses employed the Neighborhood Quality Standard algorithm [25], [54] within CLC Genomics Workbench 3.7.1, with the following user defined and default parameters: annotate consensus = yes, annotate reference = yes, create table = yes, maximum coverage = 1000X, maximum gap and mismatch count = 2, minimum average quality = 15, minimum central quality = 20, minimum coverage = 3X, minimum variant frequency = 10% or count 3, SNP analysis window = 11 bp. Custom scripts were developed to parse putative SNP locations from contigs aligned to Btau4.0, and their genomic distribution was assessed against Btau4.0.

### Functional Annotation Analyses

White-tailed deer consensus contig sequences were mapped to functional classification schemes such as Gene Ontology (GO) terms [28], KEGG pathways [29], and Swiss Prot Protein Keywords [30], via Kraken (www.krakenblast.com) which is a high throughput distributed BLAST engine based upon WND-BLAST [31] using the BLASTx algorithm and a database containing all functionally annotated protein sequences contained within Uniprot (March 2010 version), with cross reference mapping performed using DAVID [32].

### SNP Validation, Mitochondrial Phylogenetics, and Network Analysis

Nuclear SNPs were selected from white-tailed deer contigs that produced a single blastn hit (E-value≤1e-50) to one discrete bovine chromosomal coordinate (Table S7). All SNPs were selected based on the coverage and minor allele count criteria contained in Table 1, with putative SNPs predicted within or near homopolymers excluded. We also endeavored to select putative SNPs from contigs that uniquely aligned to different bovine autosomes and BTAX. Putative nuclear SNPs closely flanked by another predicted polymorphism were avoided; however, all putative mitochondrial SNPs were evaluated. Collectively, 116 putative SNPs were genotyped using the KASPar allele-specific fluorescent genotyping system (Kbiosciences; TableS7, S8) [34]. Assays exhibiting poor genotype clustering after two rounds of optimization were considered technical failures due to quality control violations [34].

All mitochondrial sequences were aligned using the ClustalW2 webserver (http://www.ebi.ac.uk/Tools/clustalw2/index.html), with manual adjustment (alignment available upon request). A Bayesian approach implemented within the program MrBayes 3.1.2 [35] was used to estimate the evolutionary history of 10 species of Cervidae by evaluating random trees using 2×10^6^ generations, GTR model with gamma-distributed rate variation, and four simultaneous Markov chains [38]. Start codons, stop codons, and overlapping regions of genes were excluded. A majority rule consensus tree rooted using the *B. taurus* outgroup was constructed and visualized within TreeView 1.6.6. For comparison, we also constructed phylogenetic trees using distance (Kimura 2 parameter), maximum composite likelihood, and maximum parsimony (transitions+transversions) with bootstrap resampling (1000 replicates; trees not shown), as referenced and implemented within the program MEGA4 [55]. All median joining haplotype networks were constructed using Network 4.5.1.0 (Fluxus Technology LTD) [34].

## Supporting Information

Figure S1 Comparative Contig Overlay with Putative SNPs. Histogram displaying the bovine chromosome locations (Btau4.0) of 19,667 blastn hits (E≤1e-50) for 18,301 white-tailed deer (WTD) sequence contigs and 6,877 putative SNPs (≥3X coverage) derived from sequencing a WTD reduced representation library (RRL).
(TIF)

Figure S2 Comparative Contig Overlay with Putative SNPs. Histogram displaying the bovine chromosome locations (Btau4.0) of 17,084 uniquely aligned (E≤1e-50) white-tailed deer (WTD) sequence contigs and 5,710 putative SNPs (≥3X coverage) derived from sequencing a WTD reduced representation library (RRL).
(TIF)

Figure S3 Comparative Contig Overlay with Putative SNPs. Histogram displaying the bovine chromosome locations (Btau4.0) of 61,553 blastn hits (E≤1e-50) for 56,084 white-tailed deer (WTD) sequence contigs and 17,813 putative SNPs (≥3X coverage) derived from sequencing a WTD reduced representation library (RRL) and random shotgun library (RSL).
(TIF)

Figure S4 Reduced representation library (RRL). White-tailed deer (WTD) genomic DNA used in the preparation of a RRL via overnight digestion of genomic DNA using *Alu*I, a 2.5% Ultra Sieve Gel (IBI Scientific) with ethidium bromide staining, and the New England Biolabs 100 bp ladder.
(TIF)

Table S1 White-tailed Deer RRL Contig Table.
(XLSX)

Table S2 White-tailed Deer RRL Informative blastn Hits (E#1e-50).
(XLSX)

Table S3 White-tailed Deer RRL Putative SNPs ($3X coverage).
(XLSX)

Table S4 White-tailed Deer RRL Functional Annotation.
(XLSX)

Table S5 White-tailed Deer RRL plus RSL (Pooled) Contig Table.
(XLSX)

Table S6 White-tailed Deer RRL plus RSL (Pooled) Informative blastn Hits (E#1e-50).
(XLSX)

Table S7 White-tailed Deer RRL plus RSL (Pooled) Putative and Validated SNPs ($3X coverage).
(XLSX)

Table S8 White-tailed Deer RRL plus RSL (Pooled) Mitochondrial SNP Table ($3X coverage).
(XLSX)

Table S9 White-tailed Deer RRL plus RSL (Pooled) Functional Annotation.
(XLSX)
