# Correction: Genome-Wide Polymorphism and Comparative Analyses in the White-Tailed Deer (*Odocoileus virginianus*): A Model for Conservation Genomics

**DOI:** 10.1371/annotation/80c6965b-ffa0-4100-8a20-3f41a08b2894

**Published:** 2011-02-03

**Authors:** Christopher M. Seabury, Eric K. Bhattarai, Jeremy F. Taylor, Ganesh G. Viswanathan, Susan M. Cooper, Donald S. Davis, Scot E. Dowd, Mitch L. Lockwood, Paul M. Seabury

Errors were introduced in the preparation of this article for publication: incorrect symbols were inserted in the legends of Tables S2, S3, S6, S7, and S8. The corrected legends are:

Table S1. White-tailed Deer RRL Contig Table (3.66Mb XLS)

Table S2. White-tailed Deer RRL Informative blastn Hits (E ≤ 1e-50; 3.00 Mb XLS)

Table S3. White-tailed Deer RRL Putative SNPs (≥ 3X coverage; 1.05 Mb XLS)

Table S4. White-tailed Deer RRL Functional Annotation (1.07 Mb XLS)

Table S5. White-tailed Deer RRL plus RSL (Pooled) Contig Table (7.99 Mb XLS)

Table S6. White-tailed Deer RRL plus RSL (Pooled) Informative blastn Hits (E ≤ 1e-50; 9.25 Mb XLS)

Table S7. White-tailed Deer RRL plus RSL (Pooled) Putative and Validated SNPs (≥ 3X coverage; 2.69 Mb XLS)

Table S8. White-tailed Deer RRL plus RSL (Pooled) Mitochondrial SNP Table (≥ 3X coverage; 0.0193 Mb XLS)

Table S9. White-tailed Deer RRL plus RSL (Pooled) Functional Annotation (2.71 Mb XLS)

