# Supplementary figures and images for: Genome-Wide Polymorphism and Comparative Analyses in the White-Tailed Deer (Odocoileus virginianus): A Model for Conservation Genomics
Source: PLoS One. 2011 Jan 19;6(1):e15811. doi: 10.1371/journal.pone.0015811 (PMC3023705; doi:10.1371/journal.pone.0015811)

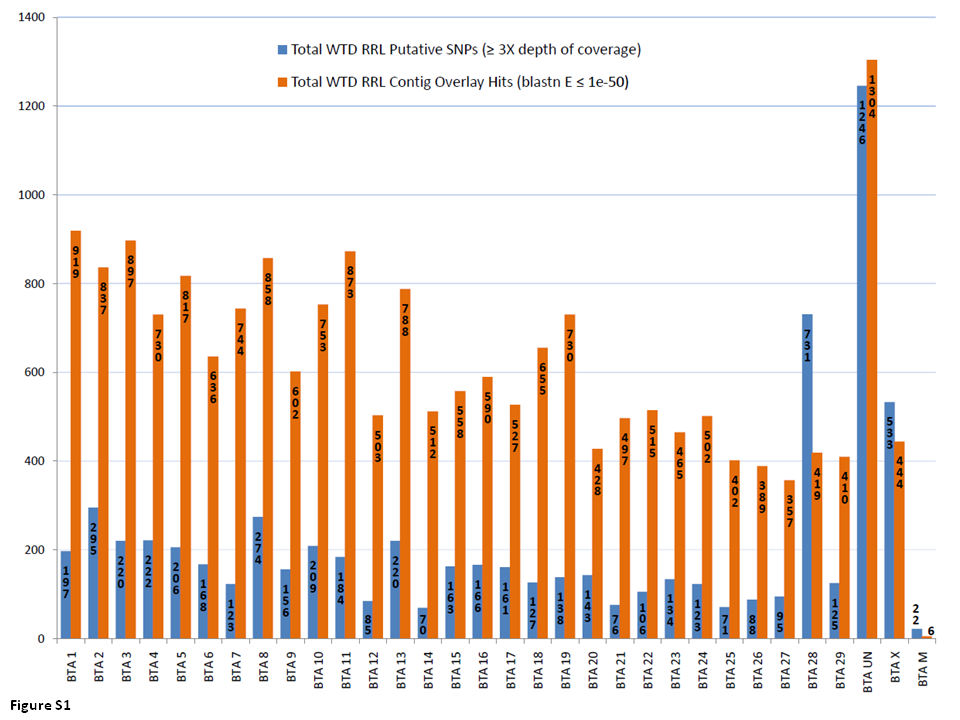

Supplement: Figure S1 — Comparative Contig Overlay with Putative SNPs. Histogram displaying the bovine chromosome locations (Btau4.0) of 19,667 blastn hits (E≤1e-50) for 18,301 white-tailed deer (WTD) sequence contigs and 6,877 putative SNPs (≥3X coverage) derived from sequencing a WTD reduced representation library (RRL). (TIF) [file pone.0015811.s001.tif]

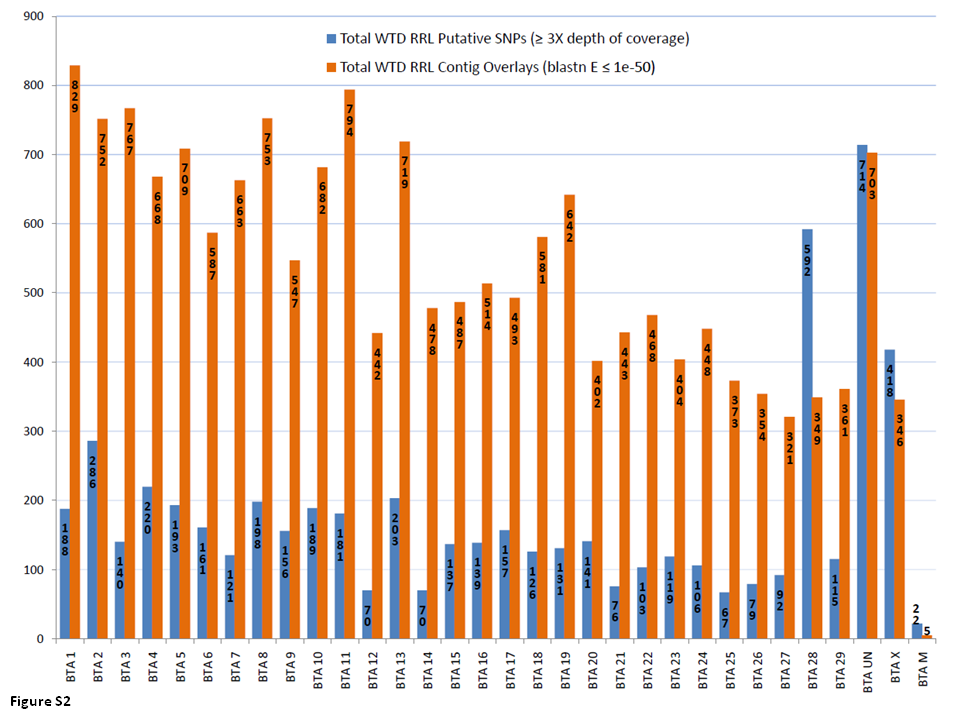

Supplement: Figure S2 — Comparative Contig Overlay with Putative SNPs. Histogram displaying the bovine chromosome locations (Btau4.0) of 17,084 uniquely aligned (E≤1e-50) white-tailed deer (WTD) sequence contigs and 5,710 putative SNPs (≥3X coverage) derived from sequencing a WTD reduced representation library (RRL). (TIF) [file pone.0015811.s002.tif]

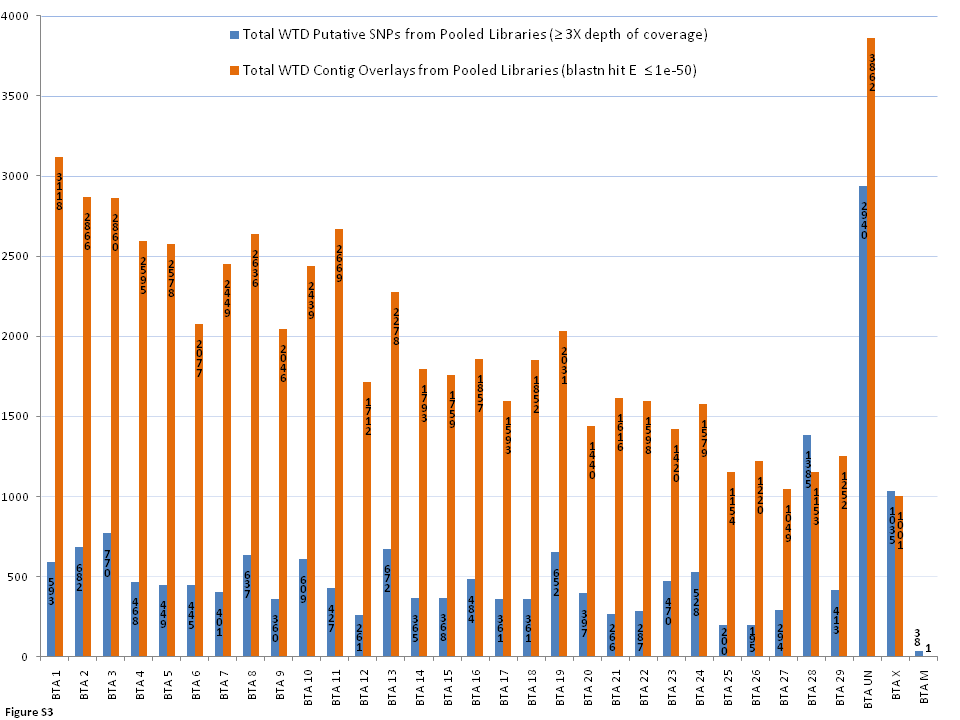

Supplement: Figure S3 — Comparative Contig Overlay with Putative SNPs. Histogram displaying the bovine chromosome locations (Btau4.0) of 61,553 blastn hits (E≤1e-50) for 56,084 white-tailed deer (WTD) sequence contigs and 17,813 putative SNPs (≥3X coverage) derived from sequencing a WTD reduced representation library (RRL) and random shotgun library (RSL). (TIF) [file pone.0015811.s003.tif]

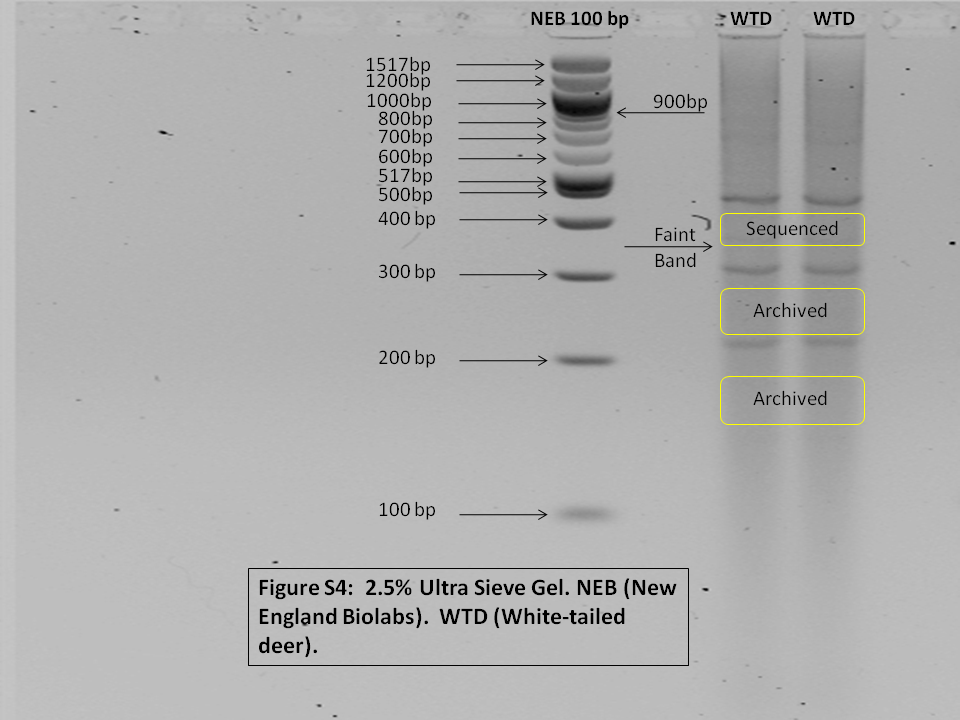

Supplement: Figure S4 — Reduced representation library (RRL). White-tailed deer (WTD) genomic DNA used in the preparation of a RRL via overnight digestion of genomic DNA using AluI, a 2.5% Ultra Sieve Gel (IBI Scientific) with ethidium bromide staining, and the New England Biolabs 100 bp ladder. (TIF) [file pone.0015811.s004.tif]
